# Initiating radical polymerization at room temperature: Why and How?

**DOI:** 10.1039/d5ra08751a

**Published:** 2026-05-19

**Authors:** Yao Fu, Xinyan Dai, Ka Leung Lam, Soham Das, Tan Zhang

**Affiliations:** a Division of Natural and Applied Sciences, Duke Kunshan University Kunshan Jiangsu 215316 China tan.zhang@dukekunshan.edu.cn

## Abstract

The energy consumption of high-temperature radical polymerizations affects the environment and human health. This review summarizes methods for initiating radical polymerization at room temperature (20–40 °C), including thermal initiation, redox initiation, interface initiation, enzymic initiation, photo initiation, and metal-mediated initiation. The energy demands for the polymerizations initiated at different temperatures were estimated. In addition to being eco-friendly, room-temperature polymerization also showed advantages in reaction safety, polymer structural control, and bio-related applications. Conducting polymerization at room temperature can be beneficial for green materials and sustainable industrial applications.

## Abstract 2

The energy consumption of high-temperature radical polymerizations affects the environment and human health.

## Graphical abstract

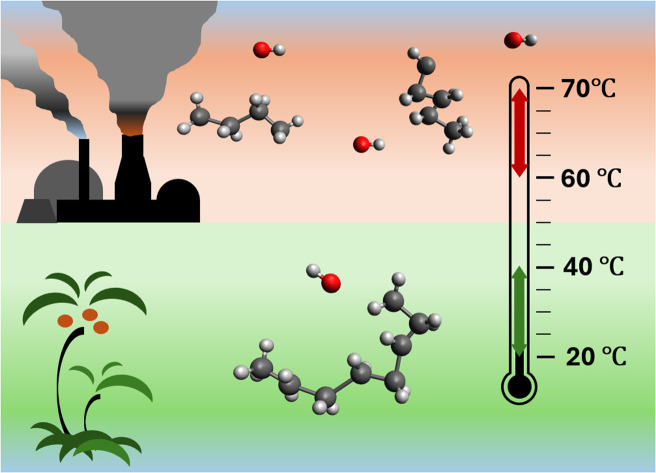

## 1 Introduction

Radical polymerization is an important reaction to produce synthetic polymers, such as polystyrene (PS), poly(methyl methacrylate) (PMMA), and many copolymers, which are widely used in packaging,^1,2^ optical lens,^3,4^ coating,^5^ electronics,^6,7^ energy storage,^8,9^ catalysis,^10,11^ and medical applications.^12,13^ Conventional radical polymerization is initiated using a thermal initiator, such as benzoyl peroxide (BPO) and 2,2′-azobisisobutyronitrile (AIBN). These initiator compounds generate free radicals at an elevated temperature, 60 °C or above, through a homolytic decomposition.^14,15^ Subsequently, the free radicals attack vinyl monomers and initiate polymer chain growth until it terminates through either primary radical termination or bimolecular coupling termination.^16,17^ It is a well-established chemical reaction and has been applied to polymer production from milligrams to tons.

Initiation at high temperatures (60 °C or above) did not draw much concern in the past decades, as it seems completely normal for a chemist to start a chemical reaction using heat. From a green chemistry perspective, high-temperature initiation, however, is not desired.^18–20^ Global annual production of synthetic polymers reached nearly 430 million tons in 2023,^21,22^ and is expected to grow by 4.2% annually till 2030.^23^ Considering the volume of polymers produced *via* free radical polymerization, the corresponding energy consumption from high-temperature initiation is significant.^24^ Energy consumption is not only an economic disadvantage but also imposes significant impacts on the environment and human health. Table 1 summarizes these impacts for the major plastic production countries. According to the principles of green chemistry, synthetic methods should be conducted at ambient temperature and pressure whenever possible.^18^ Compared with traditional initiation at high temperatures, the room-temperature approach reduces the greenhouse gas emissions by nearly half.^25^

**Table 1:** The average environmental impacts of supplying 1 kWh of electricity in major plastic production countries^a^

Country	Global warming potential (kg, CO_2_ eq.)	Fine particulate formation potential (10^−3^ kg, PM2.5 eq.)	Human carcinogenic toxicity potential (kg, 1,4-DCB)	Terrestrial acidification potential (10^−3^ kg, SO_2_ eq.)	Terrestrial ecotoxicity potential (kg, 1,4-DCB)	Freshwater ecotoxicity potential (kg, 1,4-DCB)	Marine ecotoxicity potential (kg, 1,4-DCB)
China	0.862	1.449	0.130	3.248	0.547	0.018	0.024
United States	0.491	1.354	0.049	0.907	0.259	0.017	0.022
Germany	0.479	0.237	0.060	0.677	0.249	0.026	0.034
India	1.365	3.329	0.172	4.188	0.896	0.043	0.059
Japan	0.639	0.695	0.046	1.975	0.428	0.016	0.021

a Based on Ecoinvent 3.11 (market for electricity, medium voltage) using the impact assessment method ReCiPe 2016 Midpoint (H) V1.11.

Another risk of conducting initiation at high temperature, especially for bulk polymerization, is the Trommsdorff effect. Polymer chain growth is exothermic, which releases heat during the reaction. As polymer chains are much less mobile compared with monomers, the macroscopic viscosity of the system increases with the degree of polymerization. At a certain point, the increased viscosity inhibits heat dissipation and reduces the chain termination significantly, which is the Trommsdorff effect or gel effect. The Trommsdorff effect can increase the reactor temperature significantly,^26^ which may lead to poor control of the resulting polymer structures, and more seriously, dangerous reaction safety consequences.^26,27^ The severity of the Trommsdorff effect depends on several factors associated with molecular diffusion and heat dissipation, which are related to temperature.^28^ Currently, the control of the Trommsdorff effect is achieved by using organic solvents or pumping cooling water to enhance heat dissipation. Both methods consume additional energy and risk of pollutant emission. In light of green chemistry, a green reaction should be designed to minimize the usage of organic solvents and be safe to practice.^18^

In addition to energy efficiency and safe synthesis, initiation at high temperatures also imposed many defects and limitations on the resulting polymer and polymeric materials. For example, high-temperature initiation activates side reactions, which limit the capability of the structural control.^29,30^ Colloidal templates are prone to destabilizing as the temperature increases. High-temperature initiation in colloidal templates, such as high internal phase emulsions, is at risk of phase separation and large polydispersity in pore or particle sizes.^31–33^ High-temperature initiation also makes polymerization less feasible to incorporate with temperature-sensitive molecules, such as enzymes, which denature at 40 °C or above.^14,34^ Therefore, an eco-friendly room-temperature initiation is very important and can be beneficial for many applications.

Generating radicals at room temperature or ambient conditions has been covered in several recent reviews with emphasis on emulsion polymerization,^14^ organic synthesis,^35^ or scattered in a part of other specific topics. There is a need for a comprehensive review of how to generate radicals at room temperature and the benefits from a material and environmental impact point of view. In this review, the strategies that can initiate radical polymerization at room temperature (20–40 °C) were reviewed, and the advantages of the subsequent polymerizations, the resulting polymers, and the environmental impact were discussed.

## 2 Initiation

### 2.1 Room-temperature initiator

**Scheme 1:**
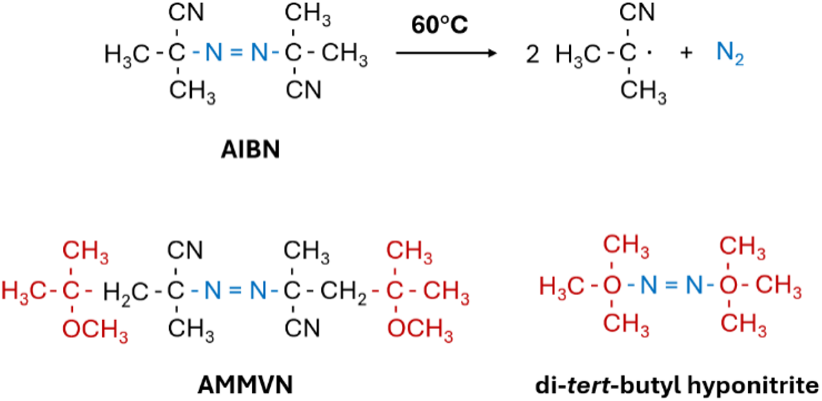
Homolytic cleavage of AIBN initiators and the structure of AMMVN and di-*tert*-butyl hyponitrite.

The most straightforward method to conduct radical polymerization at room temperature is to use an initiator that can decompose by itself at room temperature. For azo compounds, the decomposition undergoes a homolytic cleavage. For example, the two C–N bonds in an AIBN molecule break upon heating and form two radicals by releasing one nitrogen molecule (Scheme 1). To lower its decomposition temperature, the C–N bonds in azo initiators must be weakened, so the activation energy required for homolytic cleavage can be reduced. Bulky and more electronegative groups have been introduced into azo compounds for this purpose. The temperature for the 10 hour half-life for 2,2′-azobis(4-methoxy-2,4-dimethylvaleronitrile) (AMMVN or V-70) and di-*tert*-butyl hyponitrite are lowered to 30 and 40 °C, respectively.^35–37^

Trialkyl boranes, as a Lewis acid, accept electrons from oxygen readily at room temperature.^38^ The autoxidation of trialkyl borane forms an alkyl radical and a boryl peroxyl radical through β-fragmentation.^39,40^ Alkyl radicals mostly contribute to the initiation of subsequent radical reactions. Boryl peroxyl radicals are generally considered stable, but the ones with a hydrocarbon ring, such as 1-octyl-9-borabicyclononane, can react reversibly with propagating polymer radicals.^40^ It can be beneficial to control the end-group structure for the resulting polymers. As the initiation using trialkyl boranes consumes oxygen, it has been used to create an anaerobic environment for controlled radical polymerizations and regulate their kinetics.^41^

**Scheme 2:**
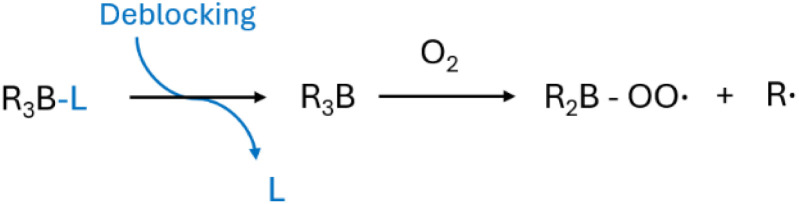
Release trialkyl borane (R_3_B) from its stabilized complex with ligand (L) and subsequent autoxidation.

Using a room-temperature thermal initiator for polymerization is relatively simple, and its kinetics can be controlled by temperature and/or oxygen. The greatest concern of using these room-temperature initiators is their extremely high reactivity at ambient conditions.^35,36^ It requires strict regulations on storage and transportation. Some strategies have been developed to stabilize these highly reactive initiators. For example, trialkyl boranes can form an oxygen-stable complex with an amine ligand, which inhibits their reaction with oxygen at ambient conditions. Before use, the amine ligand can be removed by reacting with a carbonyl compound or using irradiation (Scheme 2).^40,42–44^ Encapsulation is another method to stabilize room-temperature thermal initiators.^36^ The use of a room-temperature initiator in a polymerization is straightforward and relatively easy to practice. They can be more attractive if there is a way to store and transport them safely and conveniently.

### 2.2 Redox initiation

Redox initiation is one of the commonly used methods to initiate radical polymerization at room temperature. Redox initiation utilizes a pair of reduction and oxidation reactions to fragment the initiator through electron transfer.^45^ The activation energy for redox initiation is about 40–60 kJ mol^−1^, which is much smaller than that required to initiate thermally (100–170 kJ mol^−1^).^46^ The compound that donates electrons is called a redox-active agent, while the one that accepts electrons is an initiator.

Peroxide derivatives are mostly used as the initiator in redox initiation due to their weak O–O bond. It should be mentioned that the O–O bonds in peroxides can be cleaved homolytically through heating, making peroxides a thermal initiator. In a redox initiation system, the O–O bonds are attacked by the electron from a redox-active agent to form a radical and an anion. Other commonly used initiators for redox initiation are persulfate and disulfide compounds. Some metal complexes are also capable of generating radicals through a redox mechanism.^46^ The redox-active agents are the compounds that can be easily oxidized at ambient conditions, such as transitional metals and their organometallic complexes.^45,47–49^

**Scheme 3:**
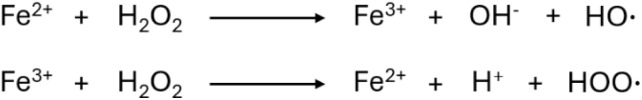
Mechanism of Fenton reaction.

The most representative redox initiation is the Fenton reaction, whose mechanism is simplified in Scheme 3.^50^ Despite only involving hydrogen peroxide and ferrous ions to start the reaction, the actual reaction pathway is complicated and remains controversial.^35,51^ The hydroxyl radicals generated from hydrogen peroxide in the Fenton reaction are highly reactive with a short lifespan.^52^ Practically, organic hydroperoxides, such as *tert*-butyl hydroperoxide and benzoyl peroxide, were used more often in initiating radical polymerization.

One of the limitations of Fenton or Fenton-like reactions is its acidic condition (pH 3–4).^50,52,53^ According to Scheme 3, Fe^2+^ ions act like a catalyst, which should be fully recovered once the reaction ends. However, the acidic condition inhibits the reaction between Fe^3+^ and hydrogen peroxide, resulting in a low efficiency in the Fe^2+^/Fe^3+^ cycle.^50^ To initiate radical reaction efficiently, a large amount of ferrous salts has to be included to maintain the concentration of ferrous ions in the system. These excess ferrous salts may contaminate the resulting polymer products and leak to the environment as a pollutant. Acidic conditions may also affect the stability of monomers and introduce side reactions.^54,55^ The most effective strategy to improve the pH tolerance for Fenton initiation is to use a heterogeneous catalyst instead of transition metal salts. Fenton-like initiation can take place in a pH-neutral solution if ferrous or ferric ions are complexed with a chelating agent or other metallic elements.^50,53,56^

In addition to traditional transition metal ions and complexes, other forms of transitional metals were also used in redox initiation.

**Fig. 1:**
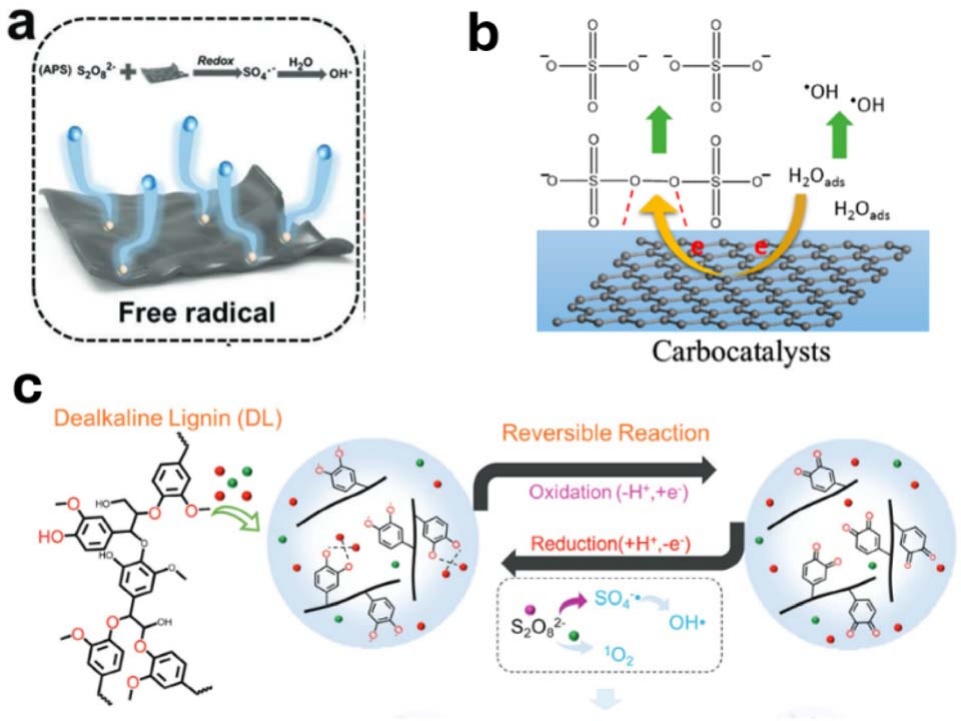
Redox initiation using (a) MXene (adapted from ref. 58 with permission from Wiley copyright 2023), (b) nanocarbon (adapted from ref. 75 with permission from ACS copyright 2015), and (c) lignin (adapted from ref. 65 with permission from Wiley copyright 2024).

MXene consists of transition metal components and therefore possesses different variable valence states.^57^ MXene can activate persulfate and initiate radical polymerization efficiently at room temperature (Fig. 1a).^58^ Incorporating copper wires can regulate the redox initiation kinetics to achieve better control of the subsequent polymerizations.^59,60^

The greatest environmental concern over redox initiation is the use of transition metals. Transition metals are hazardous to human health and the environment, and should be avoided in a green chemical process.^18,61,62^ For this purpose, several sodium salts, including sodium thiosulfate,^63^ sodium metabisulfite,^25^ sodium hydrogen sulfate,^64^ have been used to replace transition metal compounds. These alkali metal salts initiate radical polymerization in solutions or emulsions at 25–40 °C. Incorporation of lignin with alkali metal ions prompts their redox activity (Fig. 1c).^65^ The catechol groups in lignin form a stable complex with alkali metal ions, which reduces the activation energy for electron transfer. Ascorbic acid, or vitamin C, as a typical reducing agent, can activate peroxides or persulfates to initiate radical polymerization at room temperature.^66,67^ Ascorbic acid can also react with oxygen through a redox mechanism to generate radicals.^68^

Redox initiation using amine molecules is another important metal-free initiation method. In this case, the electron is transferred from the nitrogen atom in amines to the initiator through a nucleophilic attack mechanism.^35^ Diamine and triamine molecules decompose peroxide or persulfate initiators efficiently at room temperature.^67,69,70^ Pyridine, aniline, and their derivatives were also reported to initiate radical polymerization at room temperature *via* a redox mechanism.^71–73^

Interestingly, single-walled carbon nanotubes were found to be an electron donor and activate peroxides or persulfates at room temperature.^74–76^ The electron transfer is facilitated among the conjugated carbon networks and the radicals formed through the adsorbed water molecules on nanocarbon surfaces.^75^ Polar solvents and an alkaline environment promote the redox activities.^76^ Similar catalytic activities were also reported for reduced graphene oxide and mesoporous carbon.^75^ The redox activity of nanocarbon is much higher than commonly used transition metal compounds, which can be an effective strategy to avoid metal pollution for redox initiation.

Despite the effort to develop a metal-free redox initiation system, some limitations and environmental concerns are also present for the current redox initiation methods. Some substitutes for transitional metals are still considered hazardous or carcinogens.^46,49,77^ The effectiveness of redox initiation highly depends on the environment. For example, persulfates generally have better performance than peroxides in a redox system containing net anionic charges.^25^ Solubilized oxygen inhibits active radical generation significantly in a redox initiation at room temperature.^25,64,67^ Inert gas protection is required for redox initiation in most cases.

### 2.3 Interface initiation

Emulsion is a heterogeneous system consisting of an oil phase, an aqueous phase, and the oil–water interfaces stabilized by surfactants. Some monomers themselves can generate radicals in emulsions through dimerization and initiate polymerization subsequently, also known as spontaneous emulsion polymerization.^78,79^ Most dimerization occurs at 60 °C or higher and is minimized at room temperature.^79,80^ Chloroprene is the only vinyl monomer that can self-initiate and form polymers at 30 °C.^78^

Compared with oil or aqueous bulk phases, the chemical and physical environment at oil–water interfaces is very different, such as electrostatic activities,^81^ polarity gradient,^82^ thermophoretic behaviors,^83^ and interfacial tensions.^82,83^ Oil–water interfaces have been shown to be a catalytically active site for some chemical reactions, including the decomposition of initiator molecules.^14,27,84,85^ As seen in Table 2, the decomposition rate constant of AIBN at interfaces is comparable to that in the bulk phases in emulsions at 60 °C. By decreasing temperatures, the initiation of AIBN in bulk phases decreases significantly due to insufficient thermal energy provided. The radical initiation contributed by the interfaces dominates at low temperature and acts as the only radical source for polymerization conducted at 20 °C.^27^

**Table 2:** The decomposition rate constant (*k*_d_, × 10^−6^ s^−1^) for AIBN in bulk and at the interface in emulsion systems at 20, 40, and 60 °C. Reproduced from ref. 27 with permission from RSC copyright 2024

Temperature	Bulk	Interface (emulsion)	Interface (emulsion gel)
20 °C	0	0.31	0.61
40 °C	0.19	0.67	2.59
60 °C	21.97	20.61	15.03

**Fig. 2:**
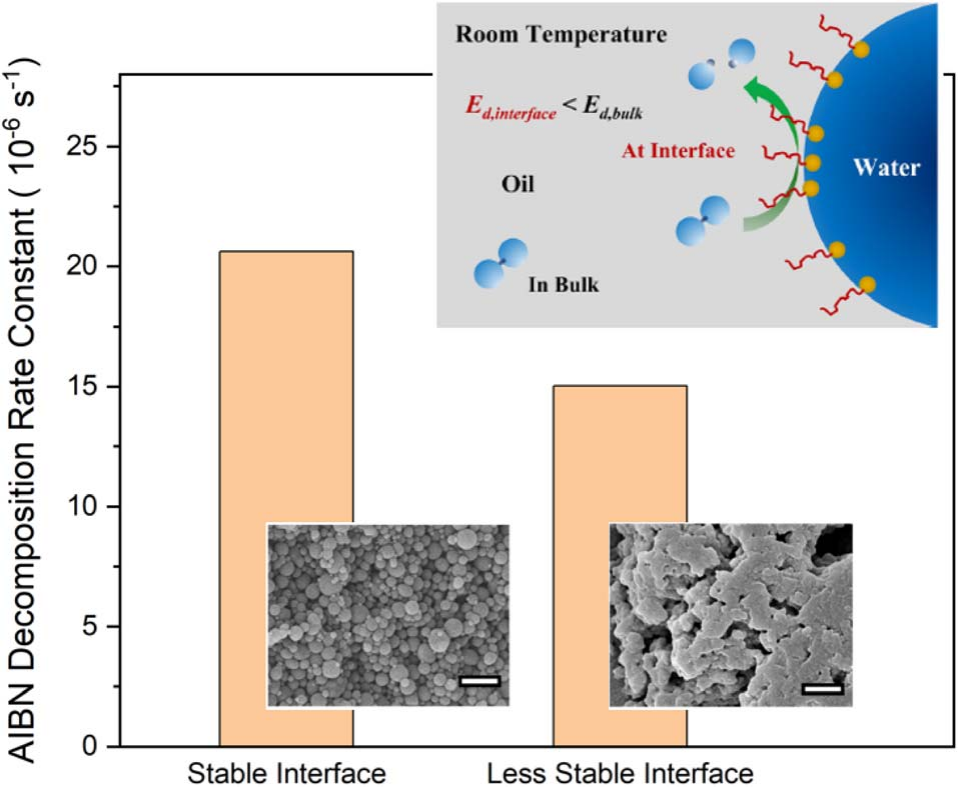
Schematic illustration of radical initiation at oil–water interfaces, and the decomposition rate constants for AIBN *versus* the stability of the interface measured at 60 °C. Figure reproduced from ref. 14 and 27 with permission from Taylor & Francis copyright 2023 and RSC copyright 2024, respectively.

The key to efficient initiation at interfaces is the stability of the oil–water interfaces.^27^ Any factors that can stabilize oil–water interfaces may facilitate interface initiation. Gel formation slows down the desorption of surfactant from oil–water interfaces, therefore stabilizing the interfaces and promoting interface initiation. An unstable oil–water interface (emulsion gel at 60 °C in Table 2) leads to slower initiation at interfaces (Fig. 2).^27^ Interfacial energy and complex formation at interfaces have been proposed as a possible mechanism.^82,86^ The surfactant type (cationic, anionic, or nonionic), initiator type (oil or water soluble), or the emulsion type (oil in water or water in oil) has little effect on the effectiveness of interface initiation.^14,82,87–89^

Interface initiation is particularly useful for initiating radical polymerization in emulsions where oil–water interfaces have already existed in the system. This strategy does not require additional chemicals or equipment for polymerization. Although inert gas protection was applied in some studies, interface initiation is not sensitive to oxygen, evidenced by high monomer-to-polymer conversion (>90%) and narrow molecular mass distribution (polydispersity index *Đ* 1.12–1.37) for the resulting polymers.^14,32,33,90^ Interface initiation can be a cost-effective method to initiate emulsion polymerization at room temperature.

### 2.4 Enzymic initiation

**Fig. 3:**
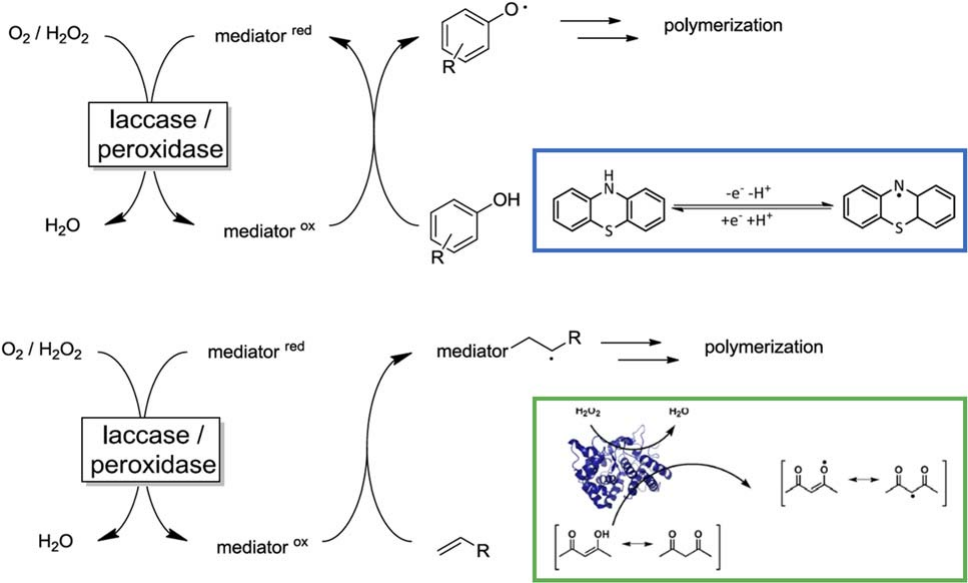
Enzymic initiation through the assistance of a mediator for radical polymerization. Reproduced from ref. 93–95 with permission from MDPI copyright 2012, ACS copyright 2015, and Elsevier copyright 2022, respectively.

Enzymes are generally considered as efficient catalysts for green chemistry.^91^ Radicals can be produced biologically through various enzymic processes.^91,92^ Two major types of enzymes used for radical initiation are peroxidases and laccases.^93,94^ The former contains an iron ion and reacts with hydrogen peroxide to generate radical species.^94^ The latter is a compound with copper ions and requires oxygen in the radical initiation.^93^ Generally, direct radical formation on monomers is not easy, so a mediator or a chain transfer agent was incorporated to facilitate the radical formation on monomers. Mediators like phenothiazine and its derivatives act as a catalyst to transfer radicals to monomers (Fig. 3a).^93^

Differently, β-diketones bond chemically to the monomer during initiation and become the end-group of the resulting polymers (Fig. 3b).^94^

Inert gas protection is not required for enzymic initiation. In fact, a proper level of oxygen is needed in enzymic initiation. Similar to other radical processes, the presence of oxygen inhibits radical polymerization significantly. Therefore, glucose oxidase is commonly used in enzymic initiation to consume excess oxygen and maintain radical activities.^91,96^ Additionally, glucose oxidase also produces hydrogen peroxide, which can help maintain the hydrogen peroxide concentration for peroxidase initiation and avoid some side reactions.^93,96^ Incorporation of glucose oxidase is particularly useful for the enzymic initiation using peroxidases. Formate oxidase was used to replace glucose oxidase in these systems to enhance the atom economy and reduce waste accumulation.^97^

Enzymic initiation is beneficial for controlled radical polymerization primarily due to the anaerobic environment created by glucose oxidase.^96^ Atom transfer radical polymerization (ATRP) requires a transition metal as a catalyst, and these ions are naturally present in enzymes. What makes it even better is that these metal ions are complexed within enzyme molecules. The possibility of metal ions leaking to the environment is minimized.^91,98^ The chain-transfer mediator approach (Fig. 3b) provides additional means to control radical transfer and helps with the structural control for reversible addition-fragmentation chain-transfer polymerization (RAFT).

**Fig. 4:**
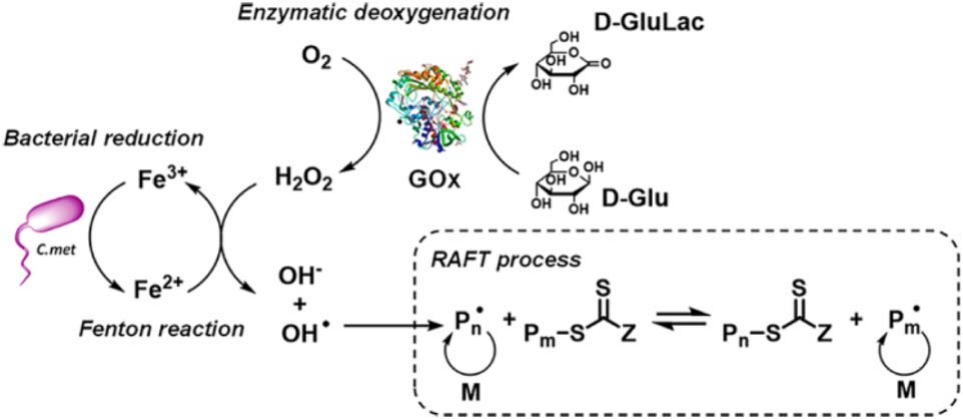
RAFT polymerization initiated by *Cupriavidus metallidurans*, adapted from ref. 99 with permission from ACS copyright 2022.

As enzymes are produced biologically, it is possible to use the reduction capability of living organisms directly to initiate radical polymerization. As shown in Fig. 4, a bacterial species of *Cupriavidus metallidurans* reduces Fe^3+^ ions effectively, therefore can participate in Fenton-like initiation with the assistance of glucose oxidase.^99^ Other microorganisms were also reported to catalyze radical generation through different mechanisms.^100^

The challenges for enzymic initiation are that its optimal reaction conditions are highly sensitive to temperature, ionic strength, solvent, monomer, and pH value.^91,101–103^ The activity of enzymes can be greatly compromised or even deactivated. After polymerization, enzymes may be trapped in the resulting polymers, and the separation is not a trivial task.^91^ The production of enzymes can be time-consuming and less cost-effective compared with other initiation methods. The stability and recycling of enzymes can be improved through immobilization onto a solid substance, but their reactivities are somewhat compromised.^104,105^

### 2.5 Photo initiation

Photo initiation utilizes irradiation, mostly ultraviolet (UV) light, to generate radicals through homolytic cleavage (type I) or hydrogen abstraction/electron transfer (type II).^106^ The diverse family of developed photo initiators is represented in Table 3, which summarizes their key characteristics. Owing to its relatively low activation energy and high rate constants, photo-initiated radical polymerization is much faster than that initiated thermally.^107,108^ It usually can be completed within several minutes, in contrast to several hours for thermal initiation. Photo initiation can be easily controlled by modulating the irradiation sources and, therefore, has been widely used in many industrial processes, such as 3D printing and thin-film coating.^109,110^ The current concerns on photo initiation include: (1) the usage of UV light, which is a risk factor for human health and also responsible for ozone production.^111^ (2) limited penetration depth inhibits the formation of thick polymer films,^112^ (3) limited applications in an aerobic environment due to oxygen sensitivity.^113^

**Table 3:** Comparison of representative photo initiators

Photo initiators	Mechanism	Advantages	Limitations
Oxime esters (OXEs)	Type I (N–O cleavage)	High thermal stability and O_2_ inhibition performance	Coloration due to photolytic residues
Acylphosphine oxides (APOs)	Type I (C–P cleavage)	Good photobleaching properties	Potential toxicity and migration issues
Phenacyl bromides (PABs)	Type I (C–Br cleavage)	Chain-end functionalization for block copolymers	Poor biocompatibility; structural modification for visible/near-infrared light absorption; side reactions
Cyanine dye/onium salt	Type II (sensitization)	Near infrared absorption and deep light penetration; high efficiency in optimized systems	Intense coloration; complex formulation
Unimolecular type II (*e.g.*, phenothiazinium sulfonium)	Type II (intramolecular)	Simplified formulation; no co-initiator migration	Complicated synthetic routes; scalability challenge; long-term stability under scrutiny

Type-I initiator is a unimolecular system where only photo-sensitive molecules with cleavable bonds. Some thermal initiators can also be used as a type-I photo initiator. For example, the C–N bonds in azo-compounds and the O–O bonds in peroxides can be cleaved upon irradiation. Therefore, when azo- and peroxide initiators were used in thermal initiation or interface initiation, the polymerization was typically conducted in a dark environment to prevent unwanted photo-initiation.^32,33^

**Scheme 4:**
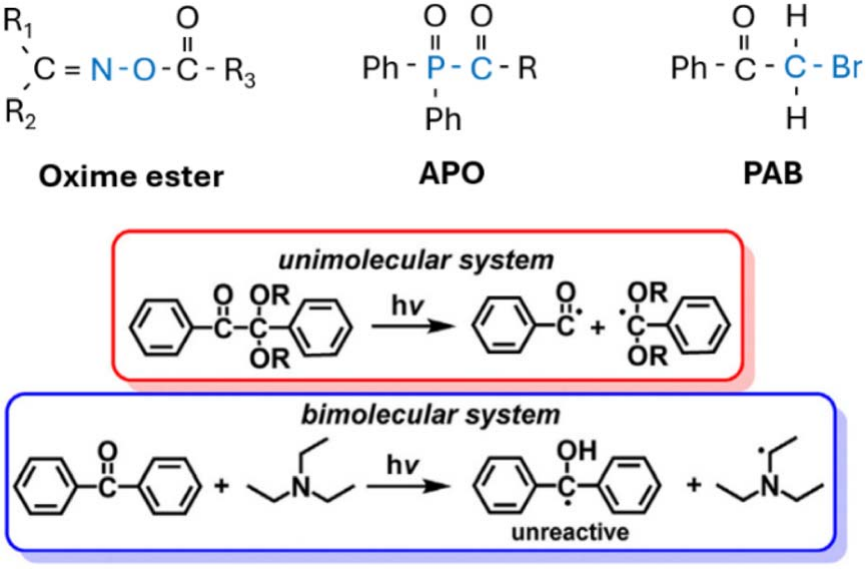
Structure of type-I photo initiator with its photocleavable bond labelled in blue, and the representative photo initiation mechanisms adapted from ref. 115 with permission from Elsevier copyright 2016.

Oxime esters (OXEs) undergo homolytic cleavage of the N–O bond from their singlet or triplet photoexcited states, and generate active alkyl radicals through decarboxylation afterwards.^114^ By introducing bulky R groups to OXEs (Scheme 4), the maximum absorption wavelength increased to the visible light region, allowing initiation using a light-emitting diode (LED) light.^116,117^ Bearing in mind that the reactivity maximum may not match the adsorption maximum.^118^ Many chromophore scaffolds have been integrated with OXEs to extend their spectral absorption beyond the UV region. These chromophores include, but are not limited to, thioxanthones,^119^ anthraquinones,^120^ carbazoles,^118,121^ chalcones,^122^ and pyrrole derivatives.^123^ Among these chromophores, electron-rich carbazole derivatives are frequently employed to achieve strong absorption under blue LED light (∼405–460 nm), offering a good balance between reactivity and synthetic accessibility.^116,120^ In contrast, chromophores with extended π-systems, such as thioxanthones and anthraquinones, enable absorption at even longer wavelengths but may introduce challenges like increased molecular weight or residual color.^120,124^

Other commonly used type-I initiators are acylphosphine oxides (APO) and phenacyl bromide (PAB). Owing to the n–π* absorption band, APO has superior photoactivity from 350–400 nm. By introducing chromophore groups, APO can be photoactive in the near-infrared region.^125^ PAB can introduce bromine end-groups to the resulting polymers, which is useful for synthesizing functional polymers.^126^ The maximum absorption for PAB derivatives can also be extended to the near-infrared region and initiate radical polymerization using an LED light source.^127^

Type-II initiators usually contain multiple components in the system, which allows intermolecular electron or hydrogen transfer. Compared with type-I initiators, type-II initiators are generally less sensitive to oxygen and easier to initiate using a long-wavelength LED source.^115^ The initiation efficiency for multicomponent ype-II initiators is limited by diffusion among different components, especially in a high-viscosity system.^128^ One-component type-II initiation is possible by introducing a hydrogen-donating group to the initiator.^113,129^ A series of chromophores or co-initiators has been introduced to onium salts, ketones, or thioxanthones, to obtain one-component initiators with a broad absorption in the visible and near infrared region.^130–134^

The extension of photoinitiation wavelengths into the near-infrared range is highly desirable for applications requiring deep light penetration, such as the curing of thick coatings and in biomedical contexts. Several dye-sensitized type-II photo-initiation systems have been developed to achieve this goal. For instance, cyanine dyes represent a powerful class, with recent work demonstrating that tris-benzo[*cd*]indole cyanine derivatives can facilitate radical polymerizations at remarkably long wavelengths up to 940 nm.^118^ The key advantage of these systems is the potential for exceptionally deep light penetration; however, primary drawbacks include their intense coloration and the need for optimized multi-component formulations to ensure high efficiency. Alternatively, unimolecular initiators chemically linked with chromophore groups offer a simplified approach. Phenothiazinium sulfonium salts exemplify this class, exhibiting near infrared photoactivity and high efficiency in free radical polymerization.^131^ Their main advantage is formulation simplicity, though their long-term stability and broad formulation compatibility represent potential limitations requiring further investigation. Another emerging class of unimolecular, metal-free initiators is the azacalixphyrins, which have been shown to operate under near-infrared light without co-initiators.^132^ This independent initiation mechanism is a significant advantage, but its structural novelty often involves challenging synthetic pathways that may pose scalability challenges.

**Scheme 5:**
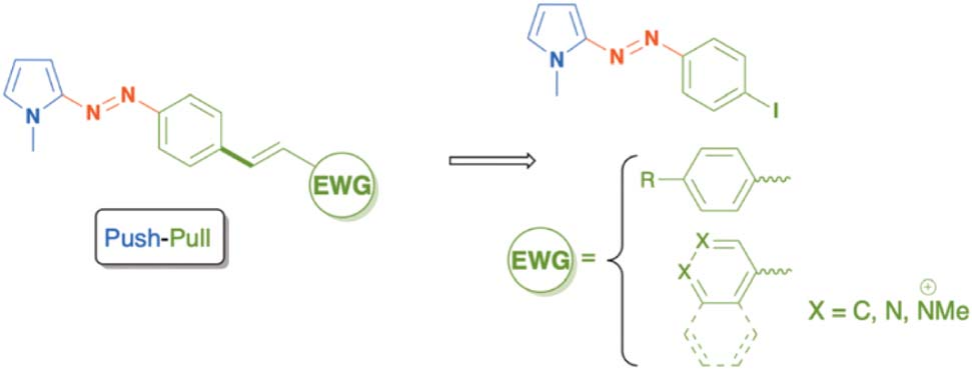
A push–pull structure based on azo-pyrrole. Adapted from ref. 137 with permission from RSC copyright 2024.

Current focus on the development of novel photo initiators from a molecular engineering perspective is introducing extended conjugates or so-called push–pull substitutes. A push–pull substitute (Scheme 5) is an asymmetric D–π–A or D–π–A–π–A′ structure bearing an electron donor (D) and electron acceptor(s) (A).^135^ Such a structure promotes intramolecular electron transfer and controls the charge relocation in the chromophores, resulting in a red shift in the absorption spectrum.^136^ Derivatives from pyrrole, triphenylamine, carbazole, and phenothiazine are commonly used as an electron donor in a push–pull structure.^118,137–139^ The challenges for push–pull structures are mainly due to their poor solubility in commonly used solvents and low initiation efficiency for thick film production. Integrating push–pull initiators into a polymer chain may enhance the solubility in the monomer phase, but the low initiation efficiency remains.^140,141^

**Fig. 5:**
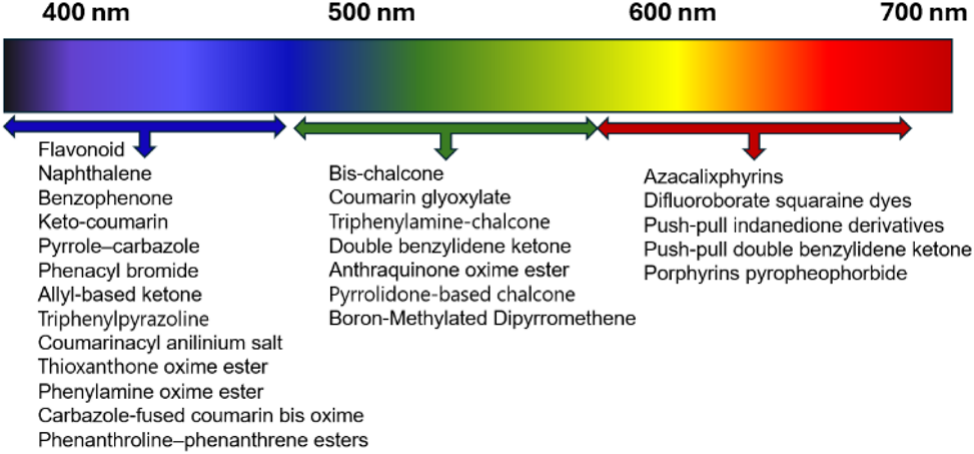
Selection of photo initiators based on their maximum absorption peak.

Photo initiation is a great room-temperature method for thin-film applications. Conducting photo initiation in an open-air environment is possible by introducing a reductive quenching cycle or a reductant,^142,143^ and the daily polymer production can reach 300 g in a flow reactor.^144^ Although the initiation using visible light or LED sources has been adequately addressed, other challenges for photo initiation remain, including high operation costs, limited penetration depth, and the use of toxic compounds.^112,145–147^ Ultrasound sonication has been integrated with photo initiation to improve penetration.^148^ Toxicity in photo initiators can be reduced by introducing biomolecules or chemical modification.^149–151^ Nevertheless, the selection of photo initiators over a wide range in the spectrum provides versatility in real-world applications (Fig. 5).

### 2.6 Metal-mediated surface initiation

**Scheme 6:**
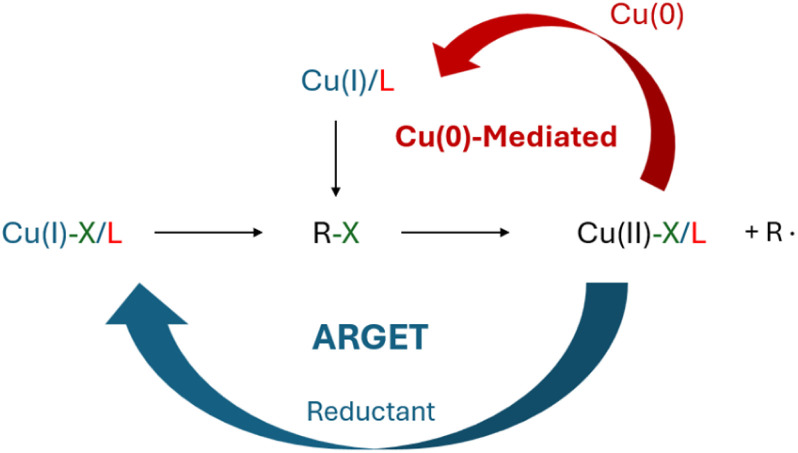
Mechanism of ARGET and Cu(0)-mediated initiation.

Transition metal elements are widely used in catalysis owing to their multiple oxidation states.^152^ It is possible that radicals can be generated on their surface, *i.e.*, surface-initiation.^153^ For example, copper (Cu) has been used to catalyze the radical generation through either Cu(0)-mediated or activators regenerated by electron transfer (ARGET) initiation, as illustrated in Scheme 6. Both bulk and nanostructured Cu can act as reducing agents, transferring electrons to an alkyl halide initiator (R–X) to generate radicals, *i.e.*, Cu(0)-mediated initiation.^153–155^ Although oxygen can inhibit polymerization by deactivating the Cu(0)/Cu(i) species and reacting with propagating chains, some research suggested that oxygen participates in the early stage of initiation.^153,156^ In an ARGET initiation system, with the assistance of a reducing agent, Cu(ii)-complexes can be reduced to Cu(i)-complexes, and the resulting Cu(i) can remove halogen from an alkyl halide initiator, leaving a radical that is ready for initiating polymerization.^157–159^ In the presence of reductants, ARGET initiation is more practical as it is more resistant to oxygen and can be conducted in an open-air environment.^158,159^ Since both initiation methods use chemical activation to generate radicals, they can be readily conducted at ambient temperature with or without other assistance.^153–156,160^

The efficiency of metal-mediated surface initiation depends on the selection of ligands. Coordinated ligands can promote electron transfer and stabilize the reaction intermediates.^161–163^ Nitrogen-based ligands are preferred in metal-mediated initiation because they are strongly coordinated to metal species and maintain a proper activation–deactivation equilibrium.^157,162^ Commonly used ligands are tris(2-pyridylmethyl)amine (TPMA) and its derivatives,^156,160,162^ tris[2-(dimethylamino)-ethyl]amine (Me_6_TREN),^164,165^ pentamethyldiethylenetriamine (PMDETA),^165^ and hexamethyltriethylenetetramine (HMTETA).^166^

The challenge to apply metal-mediated initiation is the metal residues in the resulting polymers, which are undesirable in electronics and bio-related applications. The source of Cu has been reported to affect the residue concentration.^167^ Separation of metal species from the resulting polymers can be carried out using acid washing, ion-exchange, chelation, precipitation, filtration, biphasic catalysis, or a combination of these.^168,169^ The separation procedures add negative impacts on the environment, and are still expensive and less efficient.^170^ It remains challenging to obtain the products with the concentration of metal residues that meet the requirements for electronics and biomedical applications.

### 2.7 Other initiation methods

Gamma initiation generates radicals using high-energy gamma rays, which ionize or excite monomers directly at ambient conditions.^171^ The advantage of this method is that no other chemicals or additional initiators are required for gamma initiation. Due to the high energy input to the system, side reactions in the polymerization initiated using gamma rays are inevitable. The equipment and operational costs are another factor hindering the application of gamma initiation.^172^

Ultrasound initiation utilizes the implosion of the ultrasonically generated bubbles or cavitation in water to stimulate water sonolysis. Water molecules can decompose under sonication to form hydroxyl radicals. Ultrasound initiation is limited by the equipment and is not suitable for large-scale production. A recent work applying ultrasound to initiate polymerization in a continuous flow setup may be a possible solution.^173^ Ultrasound can also be an assistance to other room-temperature initiation methods to improve their efficiency.^148^

Electrochemical initiation follows a redox mechanism but uses electrical current to trigger the reaction. The environmental impact of conducting polymerization *via* electrochemical initiation is smaller than that initiated at high temperatures.^174^ To enhance the electrochemical efficiency, an aqueous or organic solution containing a supporting electrolyte is required, but it can be difficult to recover after polymerization. Ionic liquid, as a recyclable solvent, was used as the electrolyte in electrochemical initiation.^175^ The separation of the resulting polymers from ionic liquids, however, still requires additional treatment with organic solvents, which may not be eco-friendly in this regard.^14^

## 3 Room-temperature polymerizations and the resulting polymers

### 3.1 Energy demand

Polymerization temperature is a key driver of energy consumption and associated environmental impacts. To provide a quantitative comparison across different initiation strategies, a first-order estimate of energy demand was performed for the selected polymerization systems summarized in Table 4. Those systems, which did not report their reaction time and conversions, were not included in the estimation. Energy demand was estimated using a first-order energy balance approach. Sensible heat was calculated as the energy required to raise the reaction mixture from room temperature (20 °C) to the reaction temperature, using method-specific heat capacities (bulk: 2.0, solution: 2.2, emulsion: 3.8 kJ kg^−1^ K^−1^) and monomer loadings (bulk: 1.0, solution: 0.2, emulsion: 0.3). Heat loss during reaction was approximated using a lumped heat loss coefficient (0.3 kJ h^−1^ K^−1^) and assumed constant temperature difference between the reactor and ambient conditions over the reaction time. Total energy was normalized by polymer product mass, estimated from initial monomer loading and reported conversion. Mixing and ultrasonic energy associated with the initiation stage, as well as reaction enthalpy, were not included.

**Table 4:** Comparison of polymerizations initiated at different temperatures^a^

Polymer	Method	Initiation	Temperature (°C)	Time (h)	Conversion (%)	Sensible heat (kJ kg^−1^)	Heat loss (kJ kg^−1^)	Molecular mass (kg mol^−1^)	*Đ*	Ref.
PNMVA	RAFT, bulk	V-70, thermal	35	16	61	49	118	*M*_n_ 38.6	1.33	178
PNMVA	RAFT, bulk	V-70, thermal	65	NA	31	NA	NA	*M*_n_ 28	1.27	178
PHEA	RAFT, solution	TBHP/vitamin C, redox	25	24	>99	56	182	*M*_n_ 22.2	1.11	30
PHEA	RAFT, solution	VA-044, thermal	70	2	NA	NA	NA	*M*_n_ 27.2	1.36	30
PMMA	FRP, emulsion, air	Interface	20	5 days	∼70	0	0	*M*_w_ 674	1.12	90
PMMA	FRP, emulsion, air	Interface	40	4	> 90	281	89	*M*_w_ 370	1.3	33
PMMA	FRP, emulsion, air	Interface	60	6	∼70	724	343	*M*_w_ 108	3.86	90
PS	FRP, emulsion, air	Interface	20	5 days	∼70	0	0	*M*_w_ 1661	1.37	90
PS	FRP, emulsion, air	Interface	30	24	NA	NA	NA	*M*_w_ 943	1.5	179
PS	FRP, emulsion, air	Interface	60	6	∼70	724	343	*M*_w_ 172	2.04	90
ABS	FRP, emulsion, air	Interface	20	50	90	0	0	*M*_w_ 1416	1.6	27
ABS	FRP, emulsion, air	Interface	40	10	>95	267	211	*M*_w_ 693	2.1	27
ABS	FRP, emulsion, air	Interface	60	4	88	576	182	*M*_w_ 96	2.8	27
PS	FRP, emulsion	HRP–Cu^2+^/H_2_O_2_, enzymic	r.t.	NA	84	NA	NA	*M*_n_ 309	1.57	104
PS	FRP, emulsion	HRP–Cu^2+^/H_2_O_2_, enzymic	50	NA	62	NA	NA	*M*_n_ 177	2.78	104
PMMA	RAFT, bulk	Irgacure 369, photo	40	2	53	75	23	*M*_n_ 11	1.94	180
PHEA	ATRP, solution	Ultrasonic	20	6	59	0	0	*M*_n_ 32	1.23	181

a PNMVA: poly(*N*-methyl-*N*-vinylacetamide); PHEA: poly(2-hydroxyethyl acrylate); PS: polystyrene; PMMA: poly(methyl methacrylate); ABS: acrylonitrile–butadiene–styrene copolymer; V-70: 2,2′-azobis(4-methoxy-2,4-dimethylvaleronitrile); TBHP: *tert*-butyl hydroperoxide; VA-044: 2,2′-azobis[2-(2-imidazolin-2-yl)propane]dihydrochloride; HRP: horseradish peroxidase; r.t.: room temperature; FRP: free radical polymerization; RAFT: reversible addition-fragmentation chain-transfer polymerization.

The estimated energy demand varies significantly with reaction temperature. For polymerizations conducted at or near room temperature (20–25 °C), the sensible heat requirement is negligible, and the total energy demand remains small. In contrast, reactions carried out at elevated temperatures (40–60 °C) require substantially higher energy input, primarily due to the energy required to raise the reaction mixture to the operating temperature and the associated heat loss during the reaction period. Across the systems examined, total energy demand increases markedly with temperature, reaching several hundred kJ per kg of product at 60 °C. Considering the environmental impacts associated with electricity consumption (Table 1), the reduction in energy demand achieved by room-temperature polymerization can translate directly into lower greenhouse gas emissions and reduced overall environmental burdens. These findings reinforce the importance of developing initiation strategies that operate under ambient conditions as part of a broader effort toward more sustainable polymer production. It should be noted that these estimates are based on simplified assumptions and are intended for comparative purposes. Contributions from mixing, initiation energy input (*e.g.*, ultrasonic energy), and reaction enthalpy were not included.

### 3.2 Trommsdorff effect

**Fig. 6:**
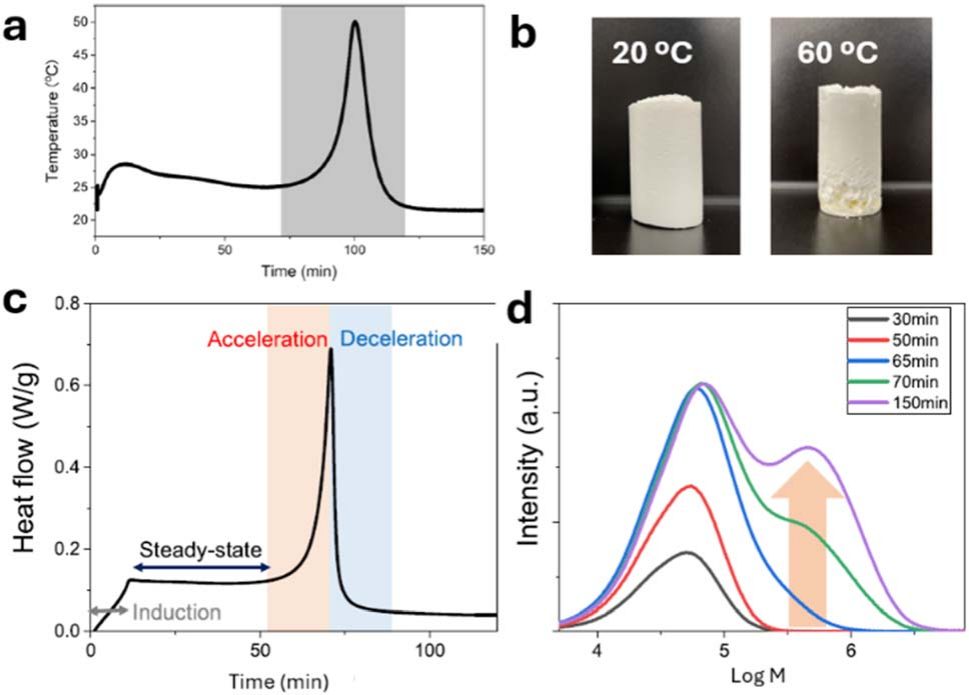
(a) Increase in temperature during the radical polymerization (adapted from ref. 177 with permission from Springer Nature copyright 2019); (b) the ABS monoliths polymerized at 20 or 60 °C (adapted from ref. 24 with permission from RSC copyright 2024); (c) heat flow during the radical polymerization; (d) molar mass of PMMA at different reaction times. (c) and (d) were adapted from ref. 176 with permission from ACS copyright 2023.

Trommsdorff effect, or gel effect, typically occurs in bulk and concentrated emulsion polymerization.^26,27^ A rapid increase in viscosity inhibits heat dissipation, evidenced by a temperature spike, during radical polymerization (Fig. 6a). The heat generated by the chain growth reaction decomposes more thermal initiators in the system, which further accelerates the polymerization and increases the viscosity dramatically.^27^ In the case of AIBN, nitrogen gas forms during initiation may also be trapped in the sample and lead to voids in the structures (Fig. 6b).^27^ The consequences of Trommsdorff effect can be dangerous as the trapped nitrogen builds up the internal pressure within the reactor. It can crack the glassware during the polymerization, and an explosion is possible.

Compared with high-temperature initiation, conducting radical polymerization at room temperature provides a low-temperature environment for faster heat dissipation. The polymerization initiated at 20 °C through interface initiation was steady without a noticeable indication Trommsdorff effect.^27^ The resulting monolith of acrylonitrile–butadiene–styrene (ABS) copolymer is uniform without voids created by Trommsdorff effect (Fig. 6b).^27^ The common practice of using an organic solvent or pumping cooling water to minimize Trommsdorff effect in radical polymerization is not necessary if the polymerization is initiated at room temperature. It not only saves energy but also avoids using toxic organic solvents. Conducting radical polymerization at room temperature can minimize Trommsdorff effect and therefore is safer to practice.

### 3.3 Molecular mass and structure

**Scheme 7:**
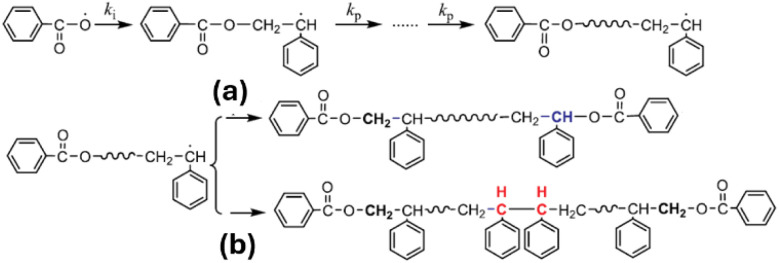
Free radical polymerization termination *via* (a) primary radical mechanism and (b) bimolecular coupling mechanism, adapted from ref. 17 with permission from Elsevier copyright 2020.

The molecular mass of polymers is mainly determined by the two competing processes, chain propagation and termination. At room temperature, termination is less favored than propagation.^32^ A propagating chain can be terminated by either reacting with a primary radical (Scheme 7a) or by combining with another propagating chain (Scheme 7b). The primary radical termination is favored in the scenario of low radical flux and low temperatures, usually leading to high molecular masses.^17^ The bimolecular termination dominates at high temperatures.^17^ It combines two propagating chains, but only occurs when two propagating chains are short enough to have their radical site exposed for termination. For room-temperature polymerizations, propagation is favored, and a polymer chain can grow to a relatively large size without encountering another propagating chain until it terminates *via* a primary radical mechanism. Therefore, polymers with higher molecular masses are possible if the polymerization is conducted at room temperature. This trend is more pronounced for free radical polymerizations (Table 4).

Chain transfer is a common side reaction that reduces molecular masses and broadens the distribution (increase in polydispersity index *Đ*) significantly.^17,182^ If the chain transfer process is well controlled, such as in a controlled radical polymerization, the molecular mass for each polymer chain can be controlled precisely, resulting in a very narrow distribution (*Đ* < 1.3).^30,96,99^ In a free radical polymerization, the probabilities for chain transfer to occur decrease with polymerization temperature.^17,183^ A relatively smaller value of *Đ* is expected for the polymers synthesized at room temperature. The temperature dependence on molecular mass distribution is less sensitive in controlled radical polymerization, as the chain transfer is controlled precisely in these polymerization systems (Table 3).

Surprisingly, the room-temperature free radical polymerization initiated using interface initiation produced homo- and copolymers with very narrow molecular mass distributions (Table 3). It should be mentioned that these free radical polymerizations were conducted in the presence of oxygen without any catalysts for controlled radical polymerization.^27,90^ The *Đ* values obtained from these free radical polymerizations are comparable to those from controlled radical polymerization. The reason is likely due to the suppression of Trommsdorff effect in the interface-initiated room-temperature polymerizations.^27^ Trommsdorff effect not only accelerates the polymerization but also creates heterogeneity in the kinetics.^176^ The *Đ* values increased by forming a bimodal distribution in molecular masses in the presence of Trommsdorff effect (Fig. 6c and d).^26^ In contrast, the polymers synthesized using interface-initiated free radical polymerizations are steady and mild. In the absence of Trommsdorff effect, the molecular mass distribution appears to be unimodal,^27,90^ resulting in a very small *Đ* value for the polymers synthesized at room temperature. If allowed, interface initiation can be an ideal candidate to replace expensive catalysts for controlled radical polymerization.

It should be emphasized that not all the free radical polymerizations initiated at room temperature can produce polymers with a narrow molecular mass distribution.^26,32,176^ The low initiation efficiency, or a relatively high ratio of monomer to radicals, allows for a steady and homogeneous environment for polymer chains to grow.^27,87,178^ Fast initiation, even at room temperature, creates too many propagating chains that favor bimolecular termination, resulting in a broader distribution. Similar results were also reported for nano-confined free radical polymerization, where the polymer chain can grow steadily without interference from other radical species.^184^

Another hint that can be deduced from these polymerizations is that the inhibition from the solubilized oxygen at room temperature may not be as strong as expected for those conducted at high temperatures. The value *Đ* of 1.1 was still achieved in the presence of oxygen.^90^ Room-temperature free radical polymerization offers an inexpensive, robust method to synthesize polymers with ultrahigh molecular mass and narrow molecular mass distribution.

Chain transfer not only terminates polymerization prematurely but also creates a tertiary radical on the polymer chain, or a midchain radical.^185^ Midchain radical may undergo branching, β-session, and termination, leading to various random chain structures.^185,186^ Acrylates are particularly prone to side reactions of chain transfer at elevated temperatures.^30,187^ Polymerization of terpenes at 70 °C produced a mixture of segments from different side reactions, while the same polymerization initiated at room temperature only yielded 1,4 addition products.^188^ Room-temperature initiation has been used in controlled radical polymerization to minimize side reactions.^189^ Conducting radical polymerization at room temperature can provide better control of the structure of the resulting polymer.

Tacticity control is difficult for free radical polymerization as monomers are randomly added to the propagating chains without any means of control, especially for the polymerization conducted at high temperatures.^190^ The tacticity of acylate polymers showed a tendency to be syndiotactic as the polymerization temperature decreased.^29,190^ For example, the segments and ester side groups on a PMMA chain are more rigid at room temperature. To react with the chain radical, the incoming MMA monomer must rearrange its ester group to the opposite position to avoid the steric repulsion from these on the chain. The steric effect enforces the growth of the PMMA chain in a syndiotactic manner. Syndiotactic-biased PMMA can be synthesized at 30–40 °C with a percentage of 〈*rr*〉 triads of 56–68%.^33,190^ The rotation of the ester side groups on a PMMA chain becomes more mobile at 55 °C or above.^191^ MMA monomers can react with the PMMA propagating chain randomly from any possible direction, resulting in an atactic PMMA chain from the high-temperature free radical polymerization.^29^

The polymerization initiated *via* photo initiation is also capable of controlling tacticity,^192^ suggesting that tacticity is primarily determined by polymerization temperature. Photo-initiated polymerization is generally faster than that initiated through other methods. In the case of acrylate polymerization, photo initiation only needs 40 min to complete the polymerization, while it took 1 hour for that initiated by a traditional thermal initiation at 100 °C.^180^ Based on limited literature on the molecular mass distribution for the polymers synthesized *via* photo initiation, the *Đ* values are generally larger than their counterparts initiated by other room-temperature methods.^134,180,193^ High radical flux in photo initiation may contribute to this phenomenon, where bimolecular coupling termination is favored.

### 3.4 Polymerization with temperature-sensitive components

**Fig. 7:**
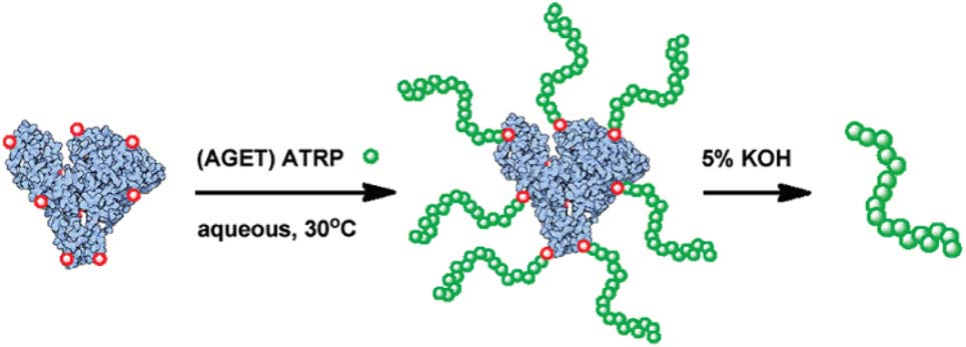
Chemical grafting on a protein with a cleavable bond at room temperature, adapted from ref. 195 with permission from ACS copyright 2012.

Biomolecules are generally sensitive to temperature, such as enzymes, which denature at 40 °C or above and lose their bioactivity.^34^ The chemical modification, encapsulation, or immobilization of these biomolecules must be conducted at room temperature. For example, poly(2-dimethylaminoethyl acrylate) is a strong binder to RNA and is usually used as a capsule shell for drug delivery. To enhance the fusion with the cancer cell membrane, the segments of poly(*N*-(3-(1*H*-imidazol-1-yl)propyl)acrylamide) need to be introduced into the capsule shell. Thanks to the room-temperature initiation *via* a copper-based catalyst, such RNA capsules can be synthesized *in situ* without compromising the bioactivities of the RNA medicine.^194^ Since precise delivery is important for drug carriers, room-temperature initiation is usually incorporated into controlled radical polymerization to synthesize target block copolymers to tune self-assembly and binding properties.^195^ Chemical modification of proteins can be done *via* a grafting-from approach with the assistance of room-temperature initiation, as demonstrated in Fig. 7.^195,196^ Non-metal room-temperature initiation methods were also used in various cases to accommodate biomolecules.^14,197^

In addition to biomolecules, introducing thermo-responsive segments to block copolymers also requires room-temperature initiation. Poly(*N*-isopropylacrylamide) failed to be copolymerized with other blocks at 70 °C due to precipitation.^30^ Room-temperature initiation plays an important role in hydrogel synthesis, which can prevent dehydration during polymerization. Redox or UV-vis light initiation has been applied to synthesize hydrogels in an aqueous environment *in situ*.^198–200^ Ammonium persulfate (APS) is usually combined with Fe^2+^ or tetramethylethylenediamine (TEMED) to initiate polymerization at room temperature for hydrogel preparation.^198,201^ As hydrogels are mostly aimed for biomedical applications, biocompatibility and toxicity must be considered when selecting initiators. For example, Glycofect was recently reported as a nontoxic option to replace TEMED in the APS/TEMED redox pair.^202^ For photo initiation, eosin-Y, camphorquinone, and some vitamin B2-based initiators are suitable to prepare biocompatible hydrogel materials.^203–205^

## 4 Conclusions and perspectives

Initiating radical polymerization at room temperature can be achieved through multiple methods, and their advantages and limitations are summarized in Table 5. Room-temperature thermal initiators are easy to use but require restricted conditions for storage and transportation. Redox initiators are versatile with many possible combinations and can be more attractive if they can be less sensitive to the reaction conditions. Interface initiation is robust and suitable for the polymerization in an emulsion system. The mechanism for interface initiation needs to be further explored. Enzymic initiation utilizes biomolecules or living microorganisms, which are green from this perspective. It can be greener if the production of the enzyme can be simplified with lower costs. Photo initiation is a mature method for thin-film applications. The penetration depth is the major limiting factor for its use in bulk production. Metal-mediated initiation was widely used for controlled radical polymerization, but the separation of metal species from the products remains challenging. Gamma radiation, ultrasound, and electrochemical initiation have also found their positions in various applications. These initiation methods have their pros and cons, and it can be challenging to improve within their own scope. It may be more effective to combine two or more initiation methods as a hybrid initiator, which may be synergistic and adapted to a robust initiation system.

**Table 5:** Comparison of room-temperature initiation methods

Initiation	Advantages	Limitations
Room-temperature initiator	Efficient without impurities	Restriction on storage and transportation
Redox	Versatile and efficient	Sensitive to the environment, metal impurities
Interface	Oxygen-tolerant and cost-effective	Emulsions, relatively slow
Enzyme	Biocompatible and more useful in aqueous systems	Sensitive to the environment, less compatible with organic solvents, and costly
Photo	Fast and efficient	Limited penetration depth
Metal-mediated	Efficient and controlled radical generation	Metal impurities

In line with the Paris Agreement, conducting polymerization at room temperature is one of the important changes we can make for a more sustainable future. The greenhouse gas and pollutant emissions can be greatly reduced if all polymerizations were conducted at room temperature. In addition to being eco-friendly, the room-temperature approach also provides a safer polymerization with better structural control of the resulting polymers. In terms of the synthesis of polymers with a narrow molecular mass distribution, free radical polymerization at room temperature can be competitive with expensive controlled radical polymerization. Conducting room temperature polymerization is not just about being green but also an important synthetic strategy for high-performance polymer materials.

Future developments of room-temperature initiation/polymerization will focus on the following areas: (1) further reducing the environmental impact by minimizing the use of transition metals, efficiently separating catalysts from the products, and reducing the use of organic solvents in the polymerization or post-polymerization treatment. (2) Improving the efficiency of the initiation to adapt more practical applications in industries. (3) Explore the applications of room-temperature initiation in various areas, including but not limited to biomedicine, total synthesis, nanomaterials, self-healing, pollutants removal, and other applications where a radical needs to be generated in ambient conditions.

## Conflicts of interest

There are no conflicts to declare.

## Data Availability

No primary research results, software or code have been included and no new data were generated or analysed as part of this review.
